# MHC binding affects the dynamics of different T-cell receptors in different ways

**DOI:** 10.1371/journal.pcbi.1007338

**Published:** 2019-09-09

**Authors:** Bernhard Knapp, P. Anton van der Merwe, Omer Dushek, Charlotte M. Deane

**Affiliations:** 1 Department of Basic Sciences, International University of Catalonia, Barcelona, Spain; 2 Department of Statistics, University of Oxford, Oxford, United Kingdom; 3 Sir William Dunn School of Pathology, University of Oxford, Oxford, United Kingdom; Imperial College London, UNITED KINGDOM

## Abstract

T cells use their T-cell receptors (TCRs) to scan other cells for antigenic peptides presented by MHC molecules (pMHC). If a TCR encounters a pMHC, it can trigger a signalling pathway that could lead to the activation of the T cell and the initiation of an immune response. It is currently not clear how the binding of pMHC to the TCR initiates signalling within the T cell. One hypothesis is that conformational changes in the TCR lead to further downstream signalling. Here we investigate four different TCRs in their free state as well as in their pMHC bound state using large scale molecular simulations totalling 26 000 ns. We find that the dynamical features within TCRs differ significantly between unbound TCR and TCR/pMHC simulations. However, apart from expected results such as reduced solvent accessibility and flexibility of the interface residues, these features are not conserved among different TCR types. The presence of a pMHC alone is not sufficient to cause cross-TCR-conserved dynamical features within a TCR. Our results argue against models of TCR triggering involving conserved allosteric conformational changes.

## Author summary

The interaction between T-cells and other cells is one of the most important interactions in the human immune system. If T-cells are not triggered major parts of the immune system cannot be activated or are not working effectively. Despite many years of research the exact mechanism of how a T-cell is initially triggered is not clear. One hypothesis is that conformational changes within the T-cell receptor (TCR) can cause further downstream signalling within the T-cell. In this study we computationally investigate the dynamics of four different TCRs in their free and bound configuration. Our large scale simulations show that all four TCRs react to binding in different ways. In some TCRs mainly the areas close to the binding region are affected while in other TCRs areas further apart from the binding region are also affected. Our results argue against a conserved structural activation mechanism across different types of TCRs.

## Introduction

**Fig 1:**
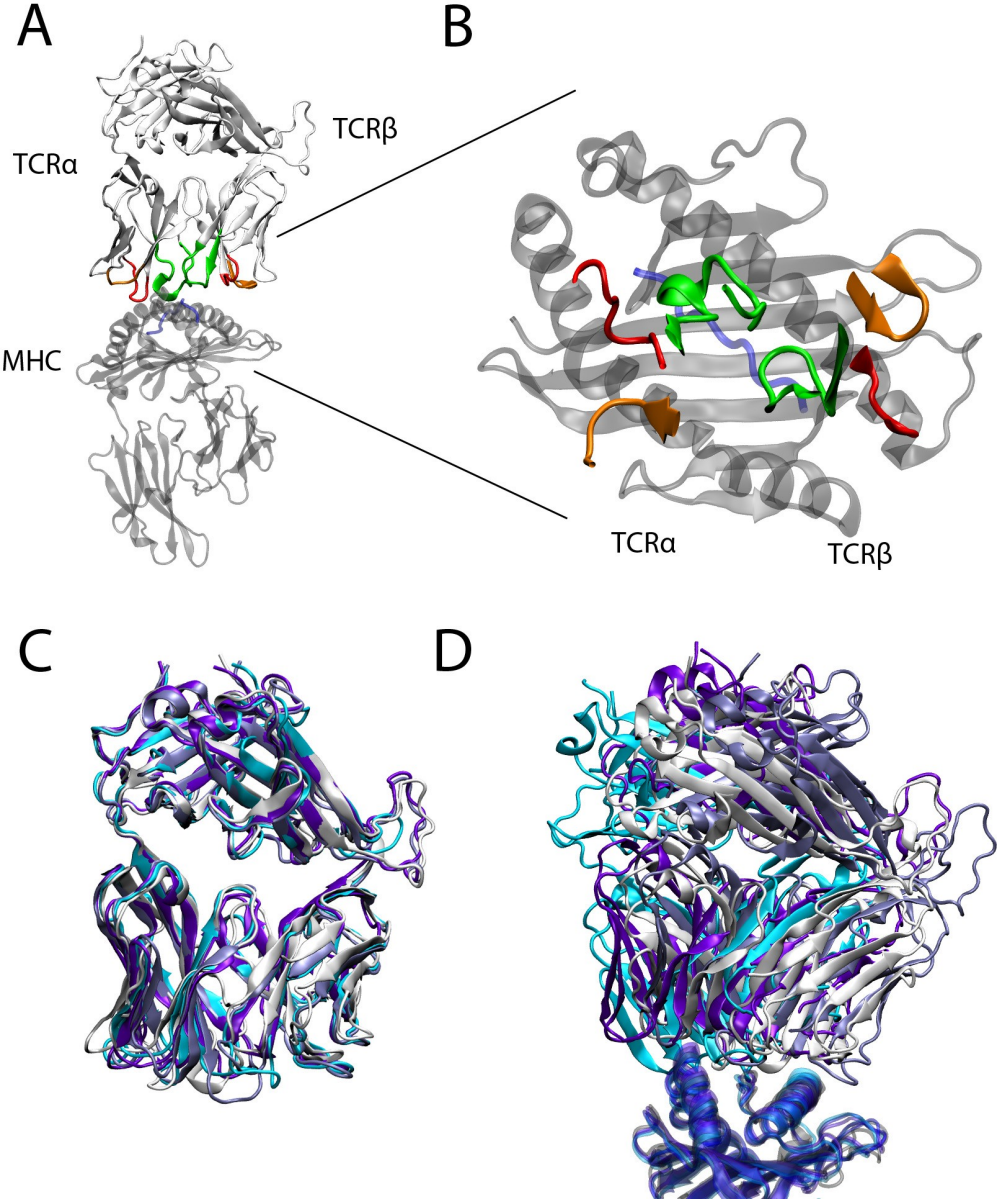
TCRpMHC structure. (A) The TCR (white) on top of the peptide (blue) and MHC (transparent grey) is shown. CDR1α (red, left), CDR2α (orange, left), CDR3α (green, left), CDR1β (red, right), CDR2β (orange, right), and CDR3β (green, right) of the TCR are in contact with the peptide/MHC complex. (B) Same as (A) but as top-view from the perspective of the TCR (only the CDRs of the TCR are shown) on the peptide/MHC (only the α_1_ and α_2_ regions of the MHC are shown). (C) Superimpositions of all four TCRs of this study. The LC13 (white), JM22 (violet), A6 (cyan) and 1G4 (light blue) TCR share a similar overall structure. (D) Same as (C) but superimposition of the MHCs (bottom). Despite their similar structure the four TCRs have slightly different binding modes on the MHCs.

The interaction between T-cell receptors (TCRs) on the surface of T-cells and peptides bound by Major Histocompatibility Complexes (MHCs) on the surface of antigen presenting cells is one of the most important processes of the adaptive immune system [1]. In the case of MHC class I molecules intracellular proteins are degraded by proteasomes into peptides, the peptides are loaded onto MHCs, and subsequently the peptide/MHC (pMHC) structures are presented on the cell surface. The TCRs of T-cells bind to pMHCs with their six hypervariable Complementarity Determining Regions (CDRs) and thereby scan the pMHC (Fig 1A and 1B). Based on this interaction further downstream signalling cascades can be activated and an immune response can be elicited against a particular antigenic peptide. The TCR/pMHC interaction is of relatively low affinity (K_D_ ~0.1–500 μM) and degenerate: One TCR can recognise multiple pMHC and one pMHC can be recognised by multiple TCRs but not every TCR can recognise every pMHC.

A long standing question has been how TCR binding to pMHC results in changes in the cytoplasmic domains of the TCR/CD3 signalling subunits (e.g. phosphorylation), a process termed TCR triggering. Several mechanisms of TCR triggering have been proposed [2], which can be grouped roughly into segregation/redistribution, aggregation, and conformational change models. Conformational change models are of two types [2]: one group, based on the observation that pMHC binding will impose a mechanical pulling force on the TCR [3], proposes that this mechanical force somehow alters the conformation of TCR relative to the membrane and/or CD3 subunits [4–6]. The second group postulates that pMHC binding is accompanied by an allosteric conformation change transduced through the TCRαβ heterodimer [7]. While there is growing support for mechanical models [8–11], evidence for ‘allosteric’ models is more equivocal (reviewed in [2]). Rossjohn and colleagues have reported a change in the AB loop of TCR Cα domain accompanying binding to agonist pMHC [7,12], but structural studies have failed to identify a conformation change conserved in all TCRs upon pMHC binding (reviewed in [2]).

The experimental studies described above relied on X-ray crystallography or NMR spectroscopy, which cannot measure dynamic changes in the TCR structure at atomistic resolution, leaving open the possibly that pMHC binding results in conserved changes in TCR dynamics. Fortunately, computational methods such as Molecular Dynamics (MD) or Monte Carlo simulations can be used to explore this possibility (reviewed in [13]). Recent approaches include TCR/pMHC interface H-bond network analysis [14,15], binding free energy [16] and detachment [17,18] simulations, effects on the MHC [19], peptide [20], and on the TCR [21] or CDR loop characterisation [22]. In a previous large scale study we analysed the same TCR/MHC in combination with 172 different peptides of known experimental immunogenicity [23]. The aim was to use simulations to seek distinct dynamical TCR behaviours for encounters with immunogenic versus non-immunogenic peptides. While no such differences were identified to be involved in discrimination, this does not rule out a role for conformational dynamics in triggering itself. In the current study we explore this by simulating four different TCRs in their agonist pMHC bound and unbound state and then look for conserved conformational features that distinguish bound from unbound TCRs. Using a total of 26 000 ns of simulation time we show that no such conserved and non-obvious features distinguish bound and unbound TCR.

## Materials & methods

### Experimental structural data

We extracted the structures of the LC13-TCR/FLRGRAYGL/HLA-B*08:01 (accession code *1MI5*), A6-TCR/LLFGYPVYV/HLA-A*02:01 (accession code *1AO7*), JM22-TCR/GILGFVFTL/HLA-A*02:01 (accession code *1OGA*), and 1G4-TCR/SLLMWITQC/HLA-A*02:01 (accession code *2BNR*) from the Protein Data Bank (PDB) [24]. Constant TCR regions were included in all simulations as we have previously shown that the constant regions are important for reliable conclusions from molecular TCR and antibody simulations [25].

The LC13 was chosen as a model system as this system has been the target of extensive MD simulations (e.g. [23,26]) due to the availability of a large number of experimentally tested mutations. Additionally we chose the A6, JM22, and 1G4 systems as they have been investigated computationally before (e.g. [14,15,18,27]) and all three TCRs bind to HLA-A*02:01 while the LC13 TCR binds HLA-B*08:01. This allows us to investigate if there are conserved TCR reaction features across MHC types as well as within HLA-A*02:01.

### Molecular simulation protocol

Eight different structures (LC13-TCR, A6-TCR, JM22-TCR, and 1G4-TCR with and without pMHC) were simulated. Each structure was immerged into a dodecahedronic simulation box filled with explicit SPC water allowing for a minimum distance of 1.2 nm between protein and box boundary. Na+ and Cl- ions were added to achieve a neutral charge and a salt concentration of 0.15 mol/litre. Protonation states were determined automatically by Gromacs [28]. Energy minimisation using the steepest descent method was applied. The systems were warmed up to 310 K using position restraints. Hydrogen atoms were replaced by virtual sites to allow for an integration step of 5 fs for the production runs [29]. Final production runs were carried out using Gromacs 4 [28] and the GROMOS 53a6 force field [30]. Parts of the LC13 simulations were taken from our previous work in [21] and [23] while parts of the A6, JM22, and 1G4 simulations were taken from [14] and [15].

Multiple replicas (identical parameter but different seeds) per simulation are important for reproducible conclusions as the results of several studies [15–17,25,31] and a systematic evaluation have shown [32]. Therefore we use 100 LC13 TCR simulation replicas at 100ns each. A boot strapping analysis of these 100 replicas (S1 Fig) shows that 10 replicas reduce the variance between replicas by about 70% while more replicas reduce the variance only slowly further (25 replicas: 80% and 50 replicas 87%). Therefore we simulated the A6, JM22, and 1G4 TCR with and without pMHC for 100 ns using 10 replicas totalling 26 μs (Table 1).

**Table 1:** Overview of simulations.

Setup	TCR	MHC	peptide	Length of one simulation	N replicas	Total simulation time
**1**	LC13	HLA-B*08	FLRGRAYGL	100 ns	100	10 000 ns
**2**	LC13	-	-	100 ns	100	10 000 ns
**3**	JM22	HLA-A*02	GILGFVFTL	100 ns	10	1 000 ns
**4**	JM22	-	-	100 ns	10	1 000 ns
**5**	A6	HLA-A*02	LLFGYPVYV	100 ns	10	1 000 ns
**6**	A6	-	-	100 ns	10	1 000 ns
**7**	1G4	HLA-A*02	SLLMWITQC	100 ns	10	1 000 ns
**8**	1G4	-	-	100 ns	10	1 000 ns
						total: 26 000 ns

### Methods of trajectory analysis: Descriptors

Trajectories were manually inspected using VMD [33] and the vmdICE-plugin [34]. Solvent accessible surface area (SASA), root mean square fluctuation (RMSF), radius of gyration (RG), hydrogen bonds (H-bonds), and distances were calculated by the GROMACS [28] modules gmx sasa, gmx rmsf, gmx gyrate, gmx hbond, and gmx distance respectively and imported into pymol/Matlab using gro2mat [35]. The H-bond networks were visualized using pyHVis3D [14].

### Comparison between TCRpMHC and TCR simulations

We used three different types of measurements to quantify the magnitude of difference between descriptors of TCRpMHC and TCR simulations (modified from our previous study [19]): Firstly, the simple difference in the mean values that is referred to as:

$$d={\bar{X}}_{TCRpMHC}-{\bar{X}}_{TCR}$$

Where ${\overset{-}{X}}_{TCRpMHC}$ and ${\overset{-}{X}}_{TCR}$ are the mean values over all frames and replicas of descriptor X (e.g. SASA or H-bonds of a region). The value *d* helps to quantify the actual magnitude of difference e.g. TCRpMHC simulations have on average 0.5 H-bonds less between their TCR chains than TCR simulations. Secondly, we normalise *d* by the range of the combined distributions excluding the highest and lowest 2.5% of the values:

$$d/r=\frac{{\bar{X}}_{TCRpMHC}-{\bar{X}}_{TCR}}{range\left({\bar{X}}_{TCRpMHC;\;2.5-97.5\%}\;\cup \;{\bar{X}}_{TCR;\;2.5-97.5\%}\right)}$$

The value *d/r* helps to quantify the magnitude of difference related to the width of the combined distributions. Thirdly, we calculate the total variation difference (*tvd*) to quantify the difference in the probability distributions:

$$TVD\;\left(f_{1},f_{2}\right)=\frac{1}{2}\;\int \left|f_{1}\left(X_{TCRpMHC}\right)-f_{2}\left(X_{TCR}\right)\right|dx$$

Where $f_{1}\left(X_{TCRpMHC}\right)$ is the normalized distribution of all TCRpMHC simulation frames and replicas and $f_{2}(X_{TCR})$ the normalized distribution of all TCR simulation frames and replicas. The *tvd* is normed between 0 and 1 where a *tvd* value of 0 represents perfect overlap of the distributions and a *tvd* value of 1 represents no overlap. In contrast to *d* and *d/r* the *tvd* does not have a sign i.e. is always positive.

### Regions of interest

We investigated the six CDR loops according to the IMGT [36] definition as extracted from the STCRDAB database [37] as well as the AB loop of the TCR α-chain which was previously hypothesised as influenced by antigen recognition of the LC13 TCR [7], the loops positioned between the variable and constant TCR domains as the linker of the β-chain (“CβFG”) was hypothesised to be important for TCR mechanosensing [10,11], αA and αΒ helices of Cβ as they were reported to be important for CD3 interaction [38], the F and C-strand of Cα as they were reported to be involved in a possible allosteric TCR signalling mechanism [39], and Cα DE and Cβ CC’ loops [40]. These regions and their sequence positon in our four different TCRs are summarised in S1 Table.

### Significance tests based on boot strapping

In order to evaluate how likely an observed descriptor difference (in *d*, *d/r* and *tvd*) would be seen by chance we performed permutation tests. We merged the n replicas of TCRpMHC simulations with the n replicas of the TCR simulations into one group of size 2n. From this 2n group we picked randomly and with repetition n members for group 1 and n members for group 2. Then we calculated *d*, *d/r* and *tvd* between group 1 and group 2. We repeated this procedure 1000 times and obtained a distribution of *d*, *d/r* and *tvd values*. Finally we determine the quartile (q) of the observed *d*, *d/r* and *tvd* between TCRpMHC and TCR within these distributions of random boot strapping samples as a indicator of significance. E.g. q = 0.98 for *d* means that 98% of all randomly created group pairs have a smaller d value between them than the d value between TCRpMHC and TCR simulations. An example for a difference in CDR1 loop distance between TCRpMHC and TCR simulations that is likely to be true for the LC13 TCR (*d* = 0.9 Å and q = 1.0 (i.e. none of the 1000 permutations had a larger difference)) and unlikely to be true the JM22 TCR (*d* = 0. 3 Å and q = 0.7 (i.e. about 300 of the 1000 permutations had a larger difference) is given in S2 Fig.

## Results

We have analysed 61 properties of our 4 TCRs in the pMHC bound and unbound state based on a total of 26 000 ns of simulation time. An overview of these results is given in Table 2 and the results are discussed in detail in the subsequent sections.

**Table 2:** Overview of analysed simulation descriptors. The difference between TCRpMHC replicas minus TCR replicas is shown. A red background indicates significantly lower values for TCRpMHC than TCR simulations while a blue background indicates significantly higher values for TCRpMHC than TCR simulations. A yellow background indicates a significant difference that has no sign (e.g. the tvd is always positive). Values that do not reach the significance threshold (top or bottom 5% of the random permutations) were set to zero (the same table without zeros is available in (S2 Table). The short-hands and method details of the left column are given in full in the respective section of the text.

	LC13	JM22	A6	1G4	LC13	JM22	A6	1G4	LC13	JM22	A6	1G4
	percDR	percDR	percDR	percDR	prcD	prcD	prcD	prcD	percTvd	percTvd	percTvd	percTvd
DIST CDR1	0,13	0,00	0,00	0,00	0,09	0,00	0,00	0,00	0,25	0,00	0,31	0,00
DIST CDR2	0,06	0,00	0,19	0,17	0,04	0,00	0,14	0,12	0,11	0,00	0,37	0,00
DIST CDR3	0,16	0,15	0,00	0,00	0,07	0,04	0,00	0,00	0,38	0,00	0,38	0,00
RG CDR1a	0,00	0,00	0,00	0,06	0,00	0,00	0,00	0,00	0,13	0,03	0,00	0,19
RG CDR1b	0,00	-0,10	0,06	0,00	0,00	-0,01	0,01	0,00	0,00	0,17	0,14	0,00
RG CDR2a	-0,06	0,00	0,12	0,00	0,00	0,00	0,01	0,00	0,14	0,00	0,23	0,00
RG CDR2b	-0,13	0,14	0,08	0,00	-0,01	0,01	0,01	0,00	0,25	0,00	0,00	0,00
RG CDR3a	-0,23	-0,18	0,26	0,33	-0,03	-0,02	0,03	0,03	0,42	0,35	0,55	0,58
RG CDR3b	-0,20	-0,15	0,00	0,13	-0,01	-0,01	0,00	0,01	0,41	0,29	0,00	0,28
RG ABloop	0,00	0,10	0,00	0,00	0,00	0,01	0,00	0,00	0,00	0,00	0,00	0,00
RG VCAlinker	0,00	0,00	0,00	0,00	0,00	0,00	0,00	0,00	0,00	0,00	0,32	0,00
RG VCBlinker	0,04	-0,21	0,00	0,00	0,00	-0,03	0,00	0,00	0,08	0,38	0,00	0,00
RG AlphaA	0,00	0,00	0,00	0,00	0,00	0,00	0,00	0,00	0,00	0,00	0,00	0,00
RG AlphaB	0,00	0,00	0,00	0,00	0,00	0,00	0,00	0,00	0,00	0,00	0,00	0,00
RG C-strand	0,00	0,00	0,00	0,00	0,00	0,00	0,00	0,00	0,00	0,00	0,00	0,00
RG F-strand	0,04	0,00	0,00	0,00	0,01	0,00	0,00	0,00	0,00	0,00	0,00	0,00
RG DE-strand	0,00	0,00	-0,12	0,00	0,00	0,00	-0,02	0,00	0,00	0,00	0,34	0,00
RG CC-strand	0,00	0,00	0,00	0,00	0,00	0,00	0,00	0,00	0,00	0,00	0,00	0,00
SASA CDR1a noMHC	0,00	0,11	0,22	0,00	0,00	0,15	0,57	0,00	0,00	0,00	0,41	0,22
SASA CDR1b noMHC	0,00	0,00	-0,10	-0,15	0,00	0,00	-0,20	-0,32	0,00	0,00	0,19	0,00
SASA CDR2a noMHC	0,00	0,00	0,11	0,00	0,00	0,00	0,21	0,00	0,00	0,00	0,00	0,00
SASA CDR2b noMHC	0,00	0,00	0,00	0,00	0,00	0,00	0,00	0,00	0,11	0,00	0,00	0,00
SASA CDR3a noMHC	-0,04	0,00	0,20	0,35	-0,13	0,00	0,57	1,37	0,10	0,00	0,34	0,58
SASA CDR3b noMHC	0,00	0,00	0,00	0,12	0,00	0,00	0,00	0,29	0,00	0,00	0,00	0,23
SASA CDR1a	-0,44	-0,31	-0,46	-0,34	-2,10	-0,72	-1,88	-0,68	0,86	0,61	0,84	0,70
SASA CDR1b	-0,27	-0,21	-0,11	-0,48	-0,54	-0,64	-0,25	-1,63	0,59	0,43	0,22	0,90
SASA CDR2a	-0,43	-0,24	-0,48	-0,28	-1,00	-0,51	-1,61	-0,74	0,85	0,52	0,89	0,54
SASA CDR2b	-0,36	-0,49	-0,13	-0,43	-1,00	-1,84	-0,27	-1,45	0,72	0,93	0,28	0,79
SASA CDR3a	-0,45	-0,52	-0,42	-0,39	-2,45	-1,92	-1,66	-1,67	0,88	0,92	0,85	0,77
SASA CDR3b	-0,53	-0,45	-0,50	-0,48	-2,62	-2,25	-2,76	-2,18	0,97	0,89	0,96	0,95
SASA ABloop	0,00	0,00	0,15	0,00	0,00	0,00	0,54	0,00	0,00	0,00	0,26	0,00
SASA VCAlinker	0,00	0,00	-0,15	0,00	0,00	0,00	-0,43	0,00	0,00	0,00	0,29	0,04
SASA VCBlinker	0,00	0,00	0,00	0,00	0,00	-0,18	0,00	0,00	0,00	0,21	0,00	0,00
SASA AlphaA	0,00	0,00	0,00	0,00	0,00	0,00	0,00	0,00	0,00	0,00	0,00	0,00
SASA AlphaB	0,00	0,00	0,00	0,00	0,00	0,00	0,00	0,00	0,00	0,00	0,00	0,00
SASA C-strand	0,00	0,00	0,00	0,00	0,00	0,00	-0,34	0,00	0,00	0,00	0,00	0,00
SASA F-strand	0,00	0,09	0,00	0,00	0,00	0,34	0,00	0,00	0,00	0,20	0,00	0,00
SASA DE-strand	0,00	0,00	0,00	0,00	0,00	0,00	0,00	0,00	0,00	0,00	0,00	0,00
SASA CC-strand	0,00	0,00	0,00	0,00	0,00	0,00	0,00	0,00	0,00	0,00	0,00	0,00
RMSF CDR1a	-0,13	0,00	0,00	-0,16	-0,03	-0,02	0,00	-0,02	0,27	0,00	0,00	0,38
RMSF CDR2a	-0,10	-0,14	0,00	0,00	-0,02	-0,02	0,00	0,00	0,18	0,00	0,00	0,00
RMSF CDR3a	-0,12	-0,13	0,00	-0,15	-0,05	-0,03	0,00	-0,04	0,21	0,31	0,00	0,00
RMSF CDR1b	-0,12	-0,19	0,00	0,00	-0,02	-0,03	0,00	0,00	0,23	0,00	0,00	0,00
RMSF CDR2b	-0,07	-0,14	0,00	0,00	-0,01	-0,03	0,00	0,00	0,16	0,00	0,00	0,00
RMSF CDR3b	-0,11	-0,18	0,00	-0,11	-0,02	-0,03	-0,03	-0,04	0,21	0,34	0,00	0,00
RMSF ABloop	0,00	0,00	0,20	0,00	0,00	0,00	0,10	0,00	0,00	0,00	0,00	0,00
RMSF VC_Alinker	0,00	0,00	0,00	0,00	0,00	0,00	0,00	0,00	0,00	0,00	0,00	0,00
RMSF VC_Blinker	0,08	-0,26	0,00	0,00	0,01	-0,03	0,00	0,00	0,00	0,47	0,00	0,00
RMSF AlphaA	0,06	0,00	0,00	0,00	0,01	0,00	0,02	0,00	0,15	0,00	0,42	0,37
RMSF AlphaB	0,06	-0,17	0,00	0,00	0,01	-0,03	0,00	-0,02	0,00	0,00	0,00	0,00
RMSF C-strand	0,00	0,00	0,00	0,00	0,00	0,00	0,00	0,00	0,00	0,00	0,00	0,00
RMSF F-strand	0,00	0,00	0,00	0,00	0,00	0,00	0,00	0,00	0,00	0,00	0,45	0,00
RMSF DE-strand	0,00	0,00	0,00	0,00	0,00	0,00	0,00	0,00	0,00	0,00	0,00	0,00
RMSF CC-strand	0,00	-0,19	0,00	-0,18	0,00	-0,05	0,00	-0,04	0,00	0,49	0,50	0,53
n of H-bonds	-0,04	-0,09	0,00	0,00	-0,51	-1,37	0,00	0,00	0,06	0,16	0,00	0,00
Change in canonical CDR shape TCRpMHC vs TCR (cohen)												
	LC13	JM22	A6	1G4								
CDR1a	0	0,21	0	0								
CDR2a	0	0	0	0								
CDR3a	0	0	0	0,29								
CDR1b	0	0	0	0								
CDR2b	0	0	0	0								
CDR3b	0	0	0	0								

### Distances between CDRs

**Fig 2:**
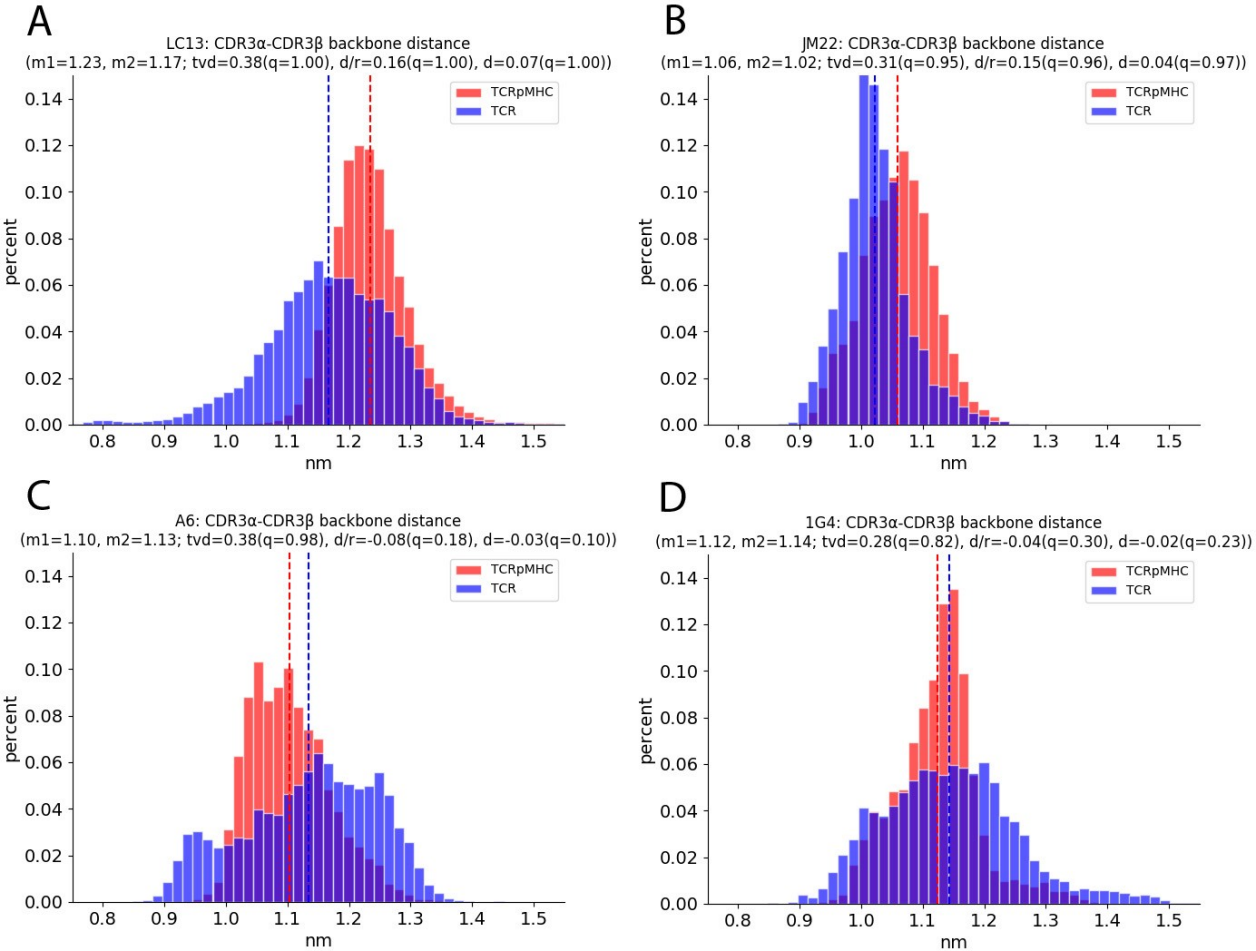
Distances between CDR3α and CDRβ as histograms. The x-axis shows the distance while the y-axis shows the occurrence of this value over all replicas and all time steps. (A) Distance of the LC13 TCR. (B) Distance of the JM22 TCR. (C) Distance of the A6 TCR. (D) Distance of the 1G4 TCR.

The distances (DIST) between the CDR loops of a TCR can be a descriptor for an opening or closing of the TCR binding interface. Especially the CDR3α and CDR3β loops that are positioned centrally over the scanned peptide (Fig 1B) and could be further apart as the pMHC presses between them or could be closer together as a result of binding interface rigidification. Our results show that the first is true for the LC13 and JM22 TCR while the A6 TCR shows a wider distribution and higher distance for TCR simulations (Fig 2). The 1G4 TCR does not show significant changes. The CDR1 and CDR2 distances also do not show patterns conserved across TCRs (Table 2).

### Radius of gyration

**Fig 3:**
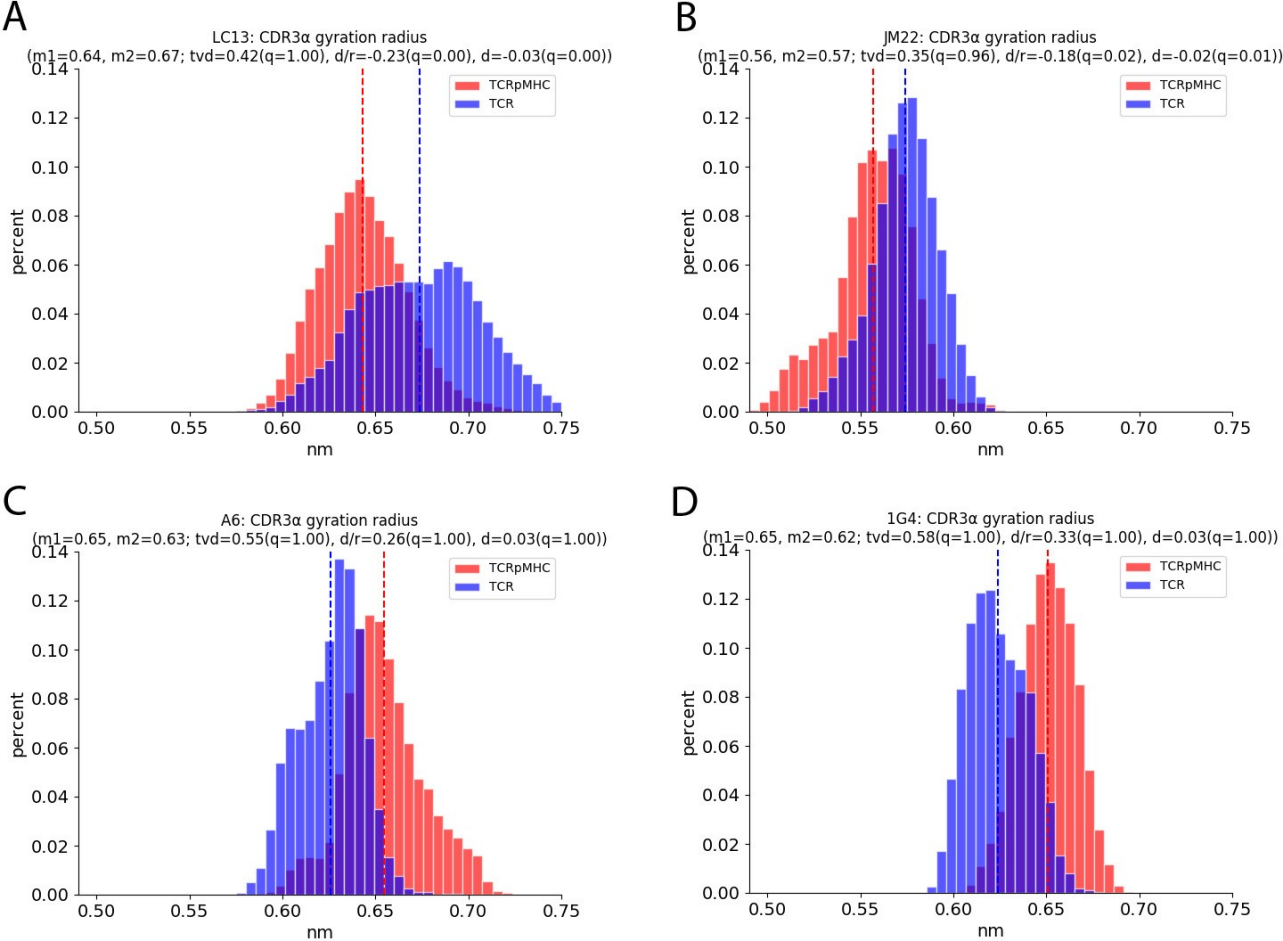
Radius of gyration of the CDR3α regions of the four TCRs. The x-axis shows the radius of gyration while the y-axis shows the occurrence of this value over all replicas and all time steps. (A) RG of the LC13 TCR. (B) RG of the JM22 TCR. (C) RG of the A6 TCR. (D) RG of the 1G4 TCR.

The radius of gyration (RG) is a proxy for the compactness of a structure. A high RG indicates a more extended structure while a low RG indicates a more compact structure. We measure the RG of all atoms of the six CDR regions as well as the ABloop, the variable/constant domain linkers and 6 further regions within the TCR constant domains to investigate if pMHC presence has “cramping” effect on any of these regions that could be involved in signalling. Several regions and TCRs show significant differences between their TCRpMHC and TCR states (Table 2); however, these are not conserved. The LC13 and JM22 TCR tend to have lower CDR RGs in their TCRpMHC simulations than in their TCR simulations while the opposite tends to be the case for A6 and 1G4. As an example we show the RG of CDR3α in Fig 3: All differences found are highly significant but they have opposing signs. In contrast the RG of the ABloop and variable/constant linkers are almost unaffected (Table 2).

### Solvent accessible surface area

**Fig 4:**
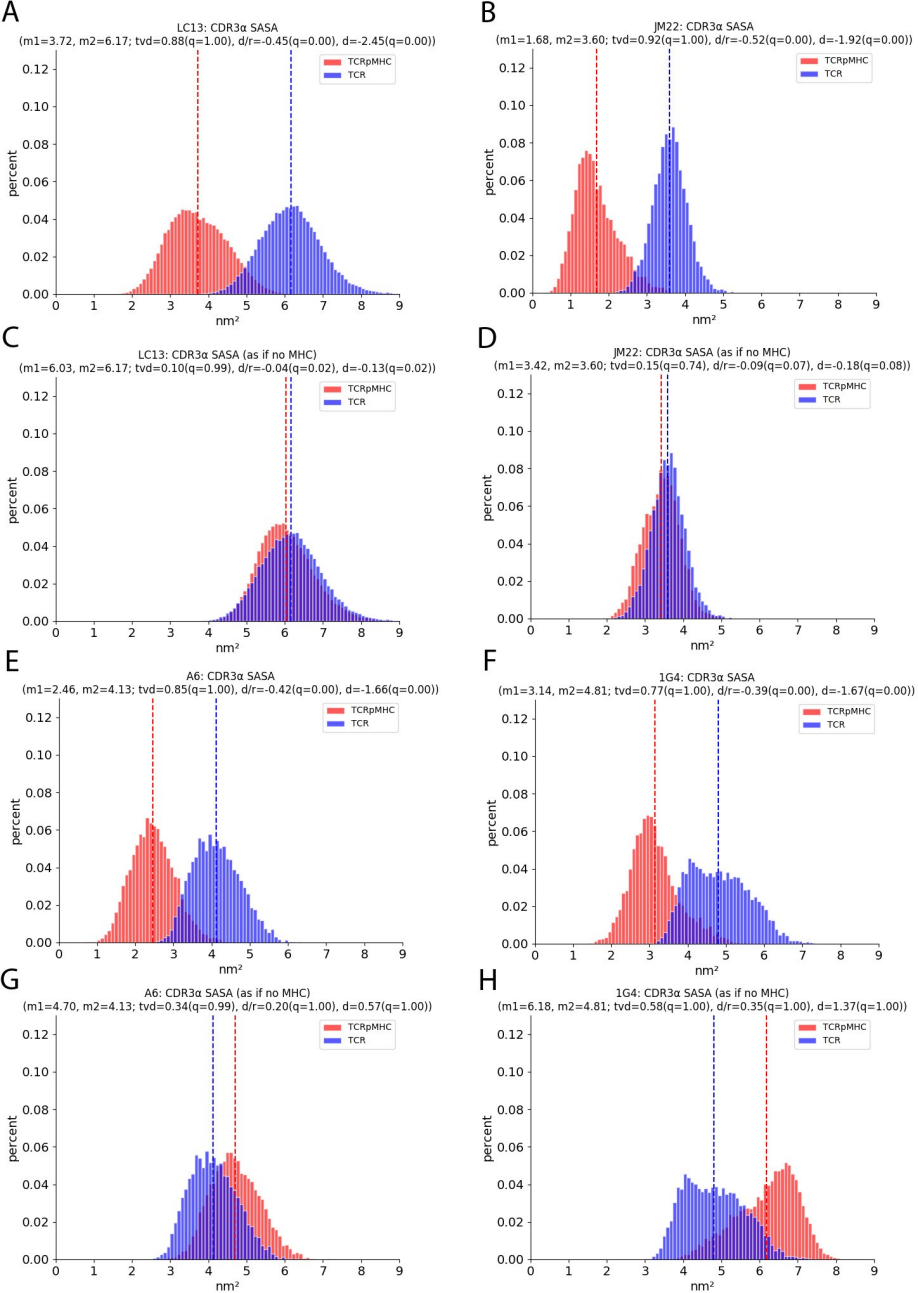
Solvent accessible surface area of CDR3α for the LC13, JM22, A6 and 1G4 TCR. The SASA measurements were taken one time with all involved structures (i.e. based on TCRpMHC for TCRpMHC and based on TCR for TCR) and a second time based only on the TCR (i.e. based on TCR for TCRpMHC and based on TCR for TCR). The x-axis shows the SASA while the y-axis shows the occurrence of this value over all replicas and all time steps. (A) SASA of CDR3α of the LC13 TCR. (B) SASA of CDR3α of the JM22 TCR. (C) SASA of CDR3α of the LC13 TCR measured without MHC. (D) SASA of CDR3α of the JM22 TCR measured without MHC. (E) SASA of CDR3α of the A6 TCR. (F) SASA of CDR3α of the 1G4 TCR. (G) SASA of CDR3α of the A6 TCR measured without MHC. (H) SASA of CDR3α of the 1G4 TCR measured without MHC.

The solvent accessible surface area (SASA) quantifies the extent that a region is exposed to solvent (here measured using the *gmx sasa* method of Gromacs). When two proteins bind the solvent accessible area of the binding interface will be reduced. This is the case for all six CDRs of all four TCRs upon pMHC binding (Table 2). The more interesting question is if there is also a change in the SASA if the SASA is measured as if no pMHC would be present for TCRpMHC simulations. i.e. is the protruding of the CDRs altered by pMHC presence? Here we obtain a picture that is partly similar to the RG-analysis. The A6 and 1G4 TCR which tend to have higher RGs in TCRpMHC simulations tend also to have increased SASAs. However, for the LC13 and JM22 TCR the reduced RG in TCRpMHC simulations seems not to lead to a decreased SASA. Fig 4 shows this effect for CDR3α.

### Root mean square fluctuation

**Fig 5:**
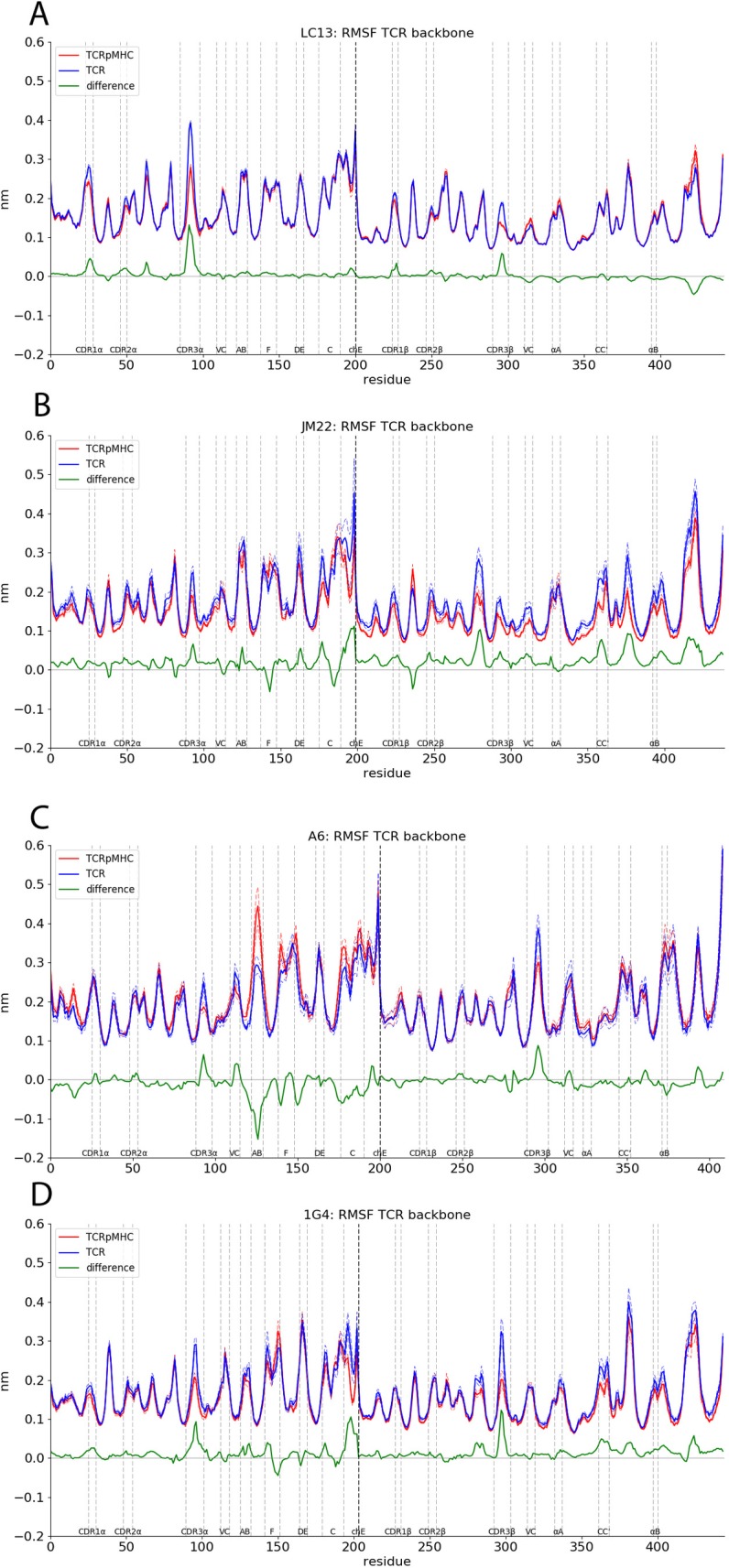
RMSF of the TCR on a per residue basis. Thick lines indicate mean values of TCRpMHC and TCR simulations while dashed lines indicate the standard error of mean. Horizontal lines mark specific regions of interest. The vertical lines at 200 indicate the border between TCR α- and β-chain. Permutation test for these regions can be found in Table 2. (A) RMSF of the LC13 TCR. (B) RMSF of the JM22 TCR. (C) RMSF of the A6 TCR. (D) RMSF of the 1G4 TCR. Please note that the RMSF curve of (A) is smoother than the others due to the use of 100 replicas instead of 10.

The root mean square fluctuation (RMSF) is an indication of how stable areas of a structure are. A potential signal transduction could be the increased or decreased flexibility of an area. We have investigated the RMSF of all TCR residues in Fig 5. Areas of special interest are marked with dashed lines. For these areas permutation tests are given in Table 2. As expected the RMSFs of the CDRs are lower in TCRpMHC simulations than in TCR simulations due to the restricted degrees of freedom in the binding interface. The changes in CDR RMSF between TCR and TCRpMHC simulations for the LC13, JM22 and 1G4 TCRs are high and mostly significant while the changes for the A6 TCR are lower and mostly not significant (compare Fig 5 and Table 2).

In contrast by far the largest observed difference in the RMSF is found in the ABloop of the A6 TCR. In TCRpMHC simulations this loop is about 50% more flexible than in TCR simulations. This is not the case for the other three TCRs. Note that the difference in ABloop arrangement between TCR and TCRpMHC was originally described for the LC13 TCR [7] and not for the A6 TCR as observed here.

### Hydrogen bonds

**Fig 6:**
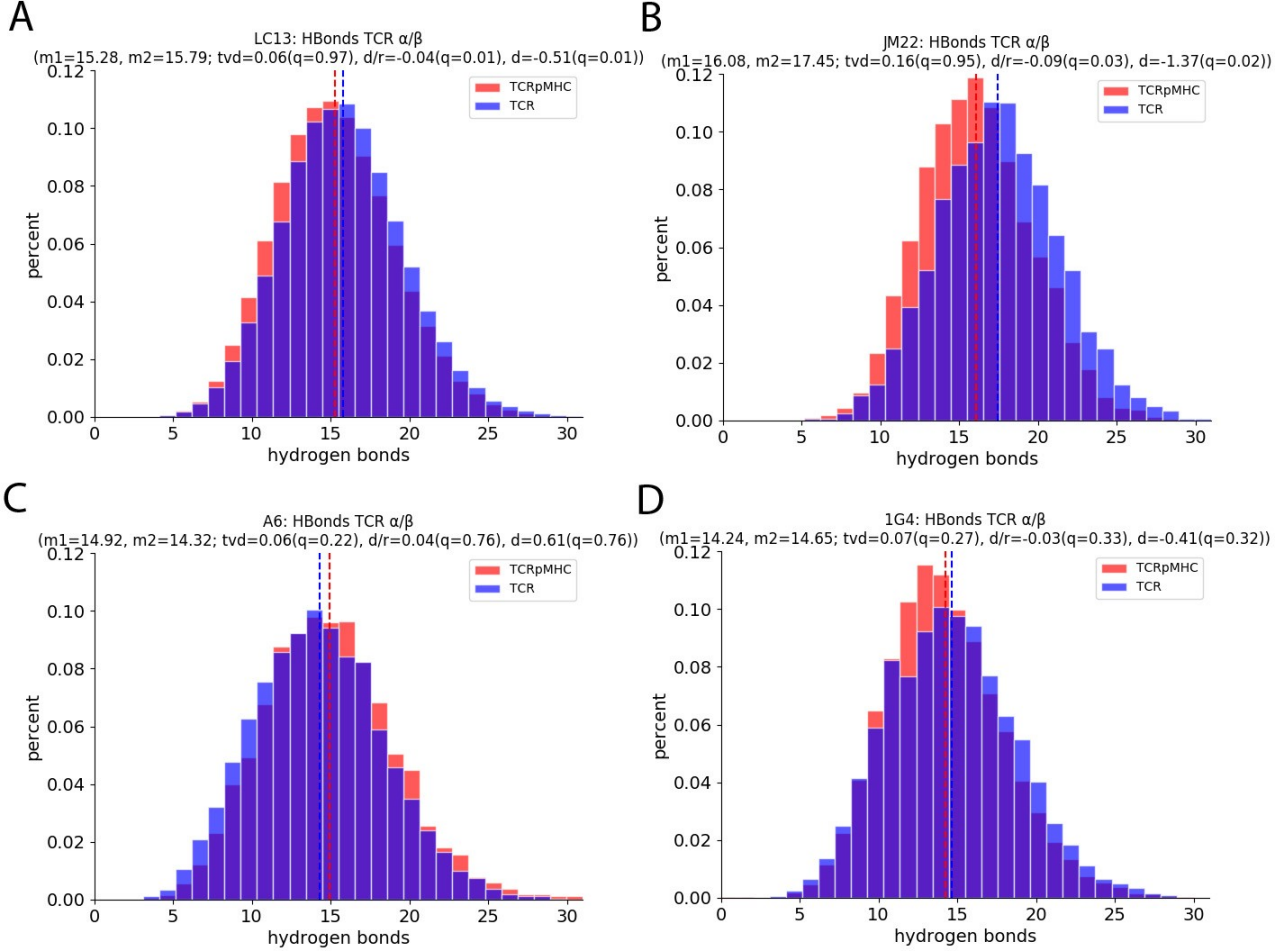
Number of H-bonds between the TCR α and β chain. The x-axis shows the number of H-bonds while the y-axis shows the occurrence of this number over all replicas and all time steps. (A) LC13 TCR. (B) JM22 TCR. (C) A6 TCR. (D) 1G4 TCR.

We also investigated the number of H-bonds between the TCR chains as a proxy for spatial re-arrangement between the TCR chains (Fig 6). For the JM22 TCR there are on average 1.37 H-bonds less between the TCR chains for TCRpMHC simulations than for TCR simulations. For the LC13 TCR this number is also slightly reduced by 0.51 H-bonds. For the A6 TCR there is a change of 0.61 H-bonds in the opposite direction but due to wider variability this number is not significant based on permutation tests (quartile 0.76). Also for the 1G4 TCR the change is insignificant.

**Fig 7:**
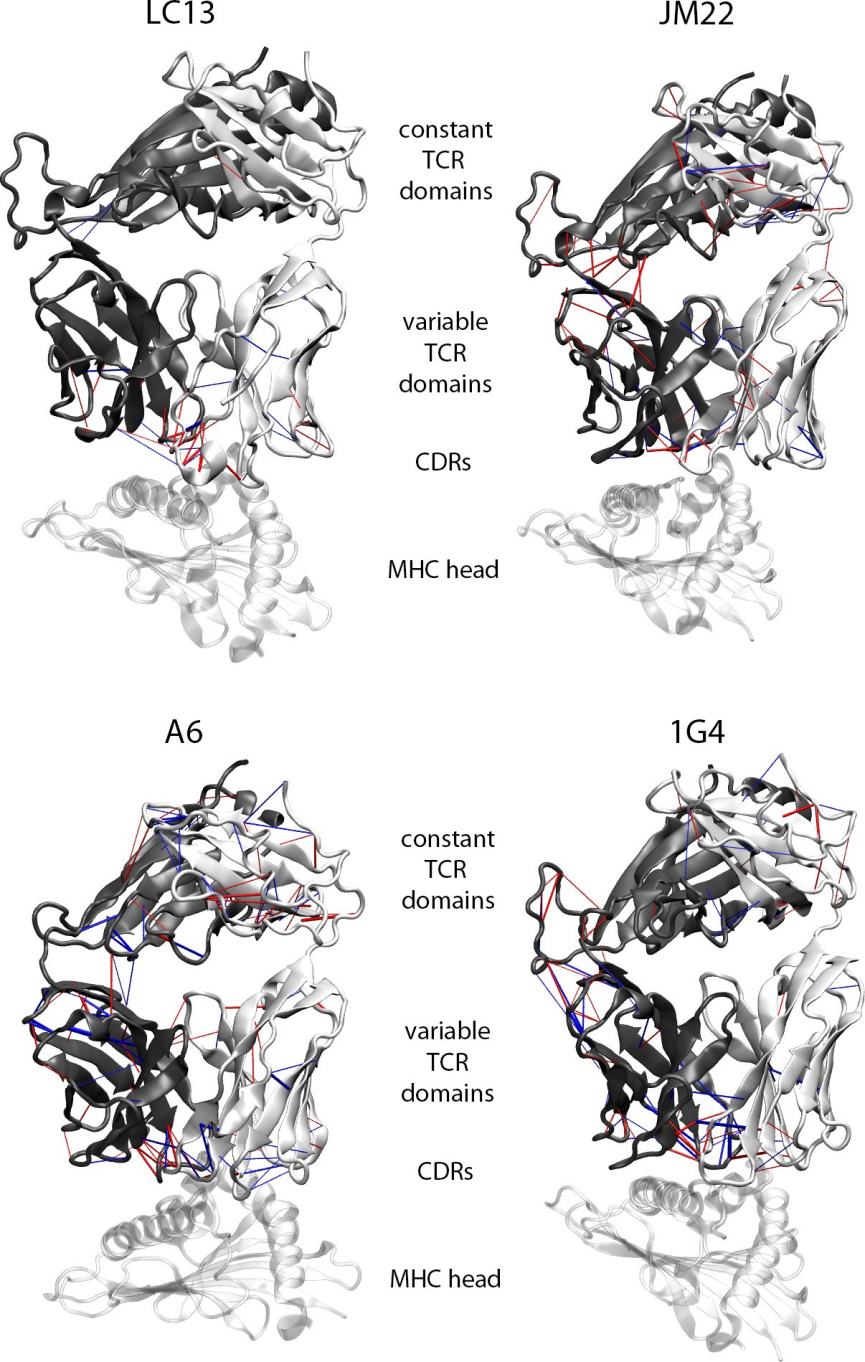
3D visualisation of H-bond patterns within the four TCRs based on PyHVis3D [14]. The radius of the cylinders is proportional to the difference between TCRpMHC simulations and TCR simulations where red cylinders indicate more H-bonds in TCRpMHC simulations while blue cylinders indicate more H-bonds in TCR simulations. Only H-bonds with a difference > 6% are shown as a boot strapping analysis (S1 Fig) indicates that theseH-bonds would not be seen by chance. TCR α-chain (white), TCR β-chain (dark grey), and peptide/MHC head (transparent white) are shown aligned by TCR orientation.

To investigate the overall H-bond network in TCRpMHC and TCR simulations we used pyHVis3D [14] which creates a three dimensional graphical representation of the H-bond distributions (Fig 7). This analysis shows different pictures for the four different TCRs. LC13 TCRpMHC simulations show an increased H-bond presence in the area around CDR3α and CDR3β as compared to LC13 TCR simulations which might indicate an interface rigidification.

This effect is also present in the JM22 TCR but it is much less pronounced than in the LC13 TCR. In the JM22 TCR the area around the linker between the variable and constant region of the TCRβ chain is mainly affected by pMHC presence causing an increased presence of H-bonds which is not observeable in the LC13 TCR.

A6 TCR simulations without pMHC show a higher H-bond frequency in the CDRs of the TCR α-chain while intra TCR chain H-bonds in this area are increased for TCRpMHC simulations. In the constant area of the TCR α-chain H-bonds dominate for TCRpMHC simulations.

Similar to JM22 also 1G4 simulations show a higher H-bonds frequency around the linker between the variable and constant region of the TCRβ chain for TCRpMHC simulations. In contrast to all other TCRs the H-bond patterns of the 1G4 TCR around the CDR loops show a very mixed picture.

Taken together this shows that there are no conserved differences in the H-bond patterns between TCR and TCRpMHC simulations.

## Discussion

We have presented an MD study of four different TCRs (LC13, JM22, A6, and 1G4) in their pMHC bound and unbound form using a total of 26 000 ns. To our knowledge this is the first study that investigates four different TCRs on such a large scale.

The most similar study was published by Hawse et al. [41] who investigated the A6 and DMF5 TCR and found a global rigidification and dampened coupling in the linker between variable and constant TCR domains upon pMHC binding using computational mutagenesis with gradient-based minimization [27] and experimental hydrogen/deuterium exchange. Also Cuendet et al. [18] investigated the detachment of the A6 and B7 TCR from HLA-A2 using about 4 000 ns of steered MD code. This study found interesting binding interface characteristics but did not address the different dynamics within the TCR in the bound and unbound form. In two studies we have previously investigated the LC13 TCR [21,23]. In one of these studies [21] we found multiple significant differences between the pMHC bound and unbound LC13 TCR. These included CDR distance distributions, CDR compactness as well as differences in the TCR hydrogen bond network. In the current study we investigated if these results hold true for other TCR/MHC combinations. Surprisingly, we obtained very different results for JM22 TCR/HLA-A*02:01 compared with LC13 TCR/ HLA-B*08:01. Thus the linker between the C and V regions of the TCR β-chain had a lower RG and RMSF for JM22 TCRpMHC simulations. Furthermore a reduced RG was seen in CDR3α and CDR3β while it was increased in CDR2β. Because LC13 and JM22 bind different MHCs, we investigated two more TCRs (A6 and 1G4) which bind the same MHC as JM22. Even when binding the same MHC type the dynamics within the TCRs vary significantly in, for example, RGs, SASAs, RMSFs (Table 2).

These differences between TCRs upon pMHC binding are consistent with our experimentally measured finding that the JM22, A6 and 1G4 TCRs have very different energetic footprints on HLA-A*02:01 [15]. Other experimental studies support the conclusions drawn in our study. With regard to changes at the binding interface, several structural studies have demonstrated local conformational changes upon TCR binding to pMHC [1], while thermodynamic and kinetic studies of several TCRs, including JM22 [42], are consistent with a reduction in conformational flexibility upon binding. With respect to conformational changes distal from the interface, while the AB-linker of the LC13 TCR has been described to differ between the X-ray structure of the HLA-B*08:01 bound and unbound structure [7], for other TCRs this finding could not be replicated. Similarly the B4.2.3 TCR was reported to be effected in its H3 loop by the binding of H2-Dd presenting a 10-mer HIV-env peptide [39]. But no further support for this being a conserved mechanism could be found in any of the other 10 TCRpMHC complex structures for which a separate TCR structure exists [39].

While our finding argue against pMHC binding inducing conformation changes through allosteric mechanism in the TCR, they remain consistent with models proposing that conformational changes are introduced by mechanical mechanisms [4,5,8–11]. For example, pulling [8,9] and shearing [10] forces have been shown to enhance TCR triggering; with the CβFG loop seemingly be affected by side-wards pulling on the TCR [11].

We conclude that TCR structural dynamics do not differ between TCR/pMHC and TCR simulations in conserved and non-obvious ways. Taken together with previous studies our findings argue against a role for allosteric conformation change models in TCR triggering. Our results are consistent with mechanical models of conformational change, as well as aggregation and kinetic-segregation models.

## Supporting information

S1 Fig Boot strapping analysis of the number of H-bonds between the two TCR chains using 100 replicas of the LC13/HLA-B8 complex. The difference (y-axis) between two randomly and with repetition chosen groups of size n (x-axis) is used. For each number of replicas per group (1 to 50) the procedure of choosing random group members from our 100 replicas was repeated 10 000 times to achieve the average difference between two groups of size n. For example two groups of one replica each differ in their mean number of H-bonds on average by 1.76+/-1.29, two groups of 10 replicas each differ by 0.53+/-0.39, and two groups of 50 replicas each differ by 0.23+/-0.18 H-bonds. For other descriptors the boot strapping curves have similar shapes.
(DOCX)

S2 Fig Permutation test explanation for a significant difference in CDR1 loop distance (LC13 TCR; top) and non-significant difference (JM22 TCR; bottom). Left: distribution of values. Right: distribution of the permutation tests.
(DOCX)

S3 Fig Comparison between RMSF values of the simulations and experimental B-factors. RMSF and B-factors where normalised by subtracting the mean and dividing by the standard deviation in order to be on the same scale for plotting. This does not change the value of the correlation coefficient given in the title of the plots. (A) LC13 TCR, (B) JM22 TCR, (C) A6 TCR, (D) 1G4 TCR.
(DOCX)

S1 Table Regions of interest of the four TCRs based on superimposition. (DOCX)

S2 Table Same as Table 2 but showing and colouring also non-significant values. (DOCX)
