# Supplementary material for: MHC binding affects the dynamics of different T-cell receptors in different ways
Source: PLoS Comput Biol. 2019 Sep 9;15(9):e1007338. doi: 10.1371/journal.pcbi.1007338 (PMC6752857; doi:10.1371/journal.pcbi.1007338)
Supplement: S1 Fig — The difference (y-axis) between two randomly and with repetition chosen groups of size n (x-axis) is used. For each number of replicas per group (1 to 50) the procedure of choosing random group members from our 100 replicas was repeated 10 000 times to achieve the average difference between two groups of size n. For example two groups of one replica each differ in their mean number of H-bonds on average by 1.76+/-1.29, two groups of 10 replicas each differ by 0.53+/-0.39, and two groups of 50 replicas each differ by 0.23+/-0.18 H-bonds. For other descriptors the boot strapping curves have similar shapes. (DOCX) [file pcbi.1007338.s001.docx]

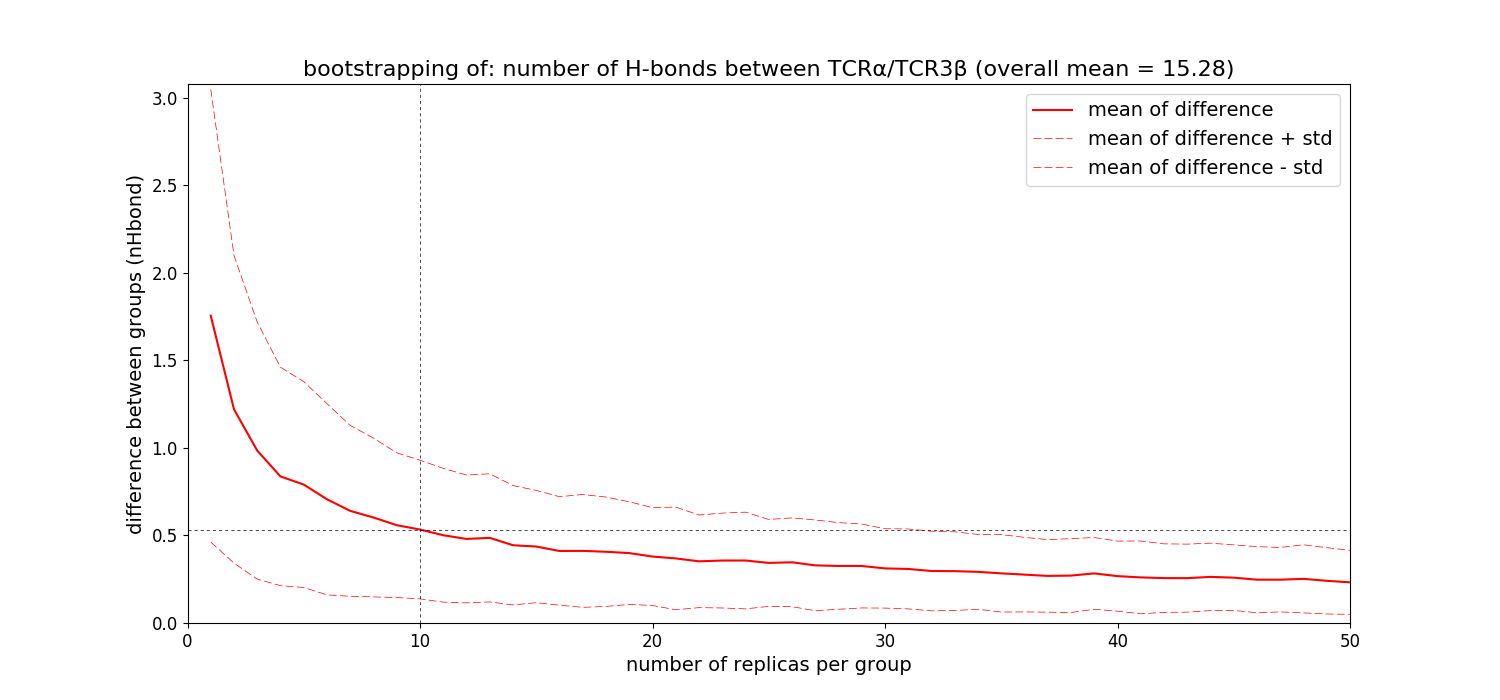


Figure S 1: Boot strapping analysis of the number of H-bonds between the two TCR chains using 100 replicas of the LC13/HLA-B8 complex. The difference (y-axis) between two randomly and with repetition chosen groups of size n (x-axis) is used. For each number of replicas per group (1 to 50) the procedure of choosing random group members from our 100 replicas was repeated 10 000 times to achieve the average difference between two groups of size n. For example two groups of one replica each differ in their mean number of H-bonds on average by 1.76+/-1.29, two groups of 10 replicas each differ by 0.53+/-0.39, and two groups of 50 replicas each differ by 0.23+/-0.18 H-bonds.
For other descriptors the boot strapping curves have similar shapes (data not shown).
