# Supplementary material for: MHC binding affects the dynamics of different T-cell receptors in different ways
Source: PLoS Comput Biol. 2019 Sep 9;15(9):e1007338. doi: 10.1371/journal.pcbi.1007338 (PMC6752857; doi:10.1371/journal.pcbi.1007338)
Supplement: S2 Fig — Left: distribution of values. Right: distribution of the permutation tests. (DOCX) [file pcbi.1007338.s002.docx]

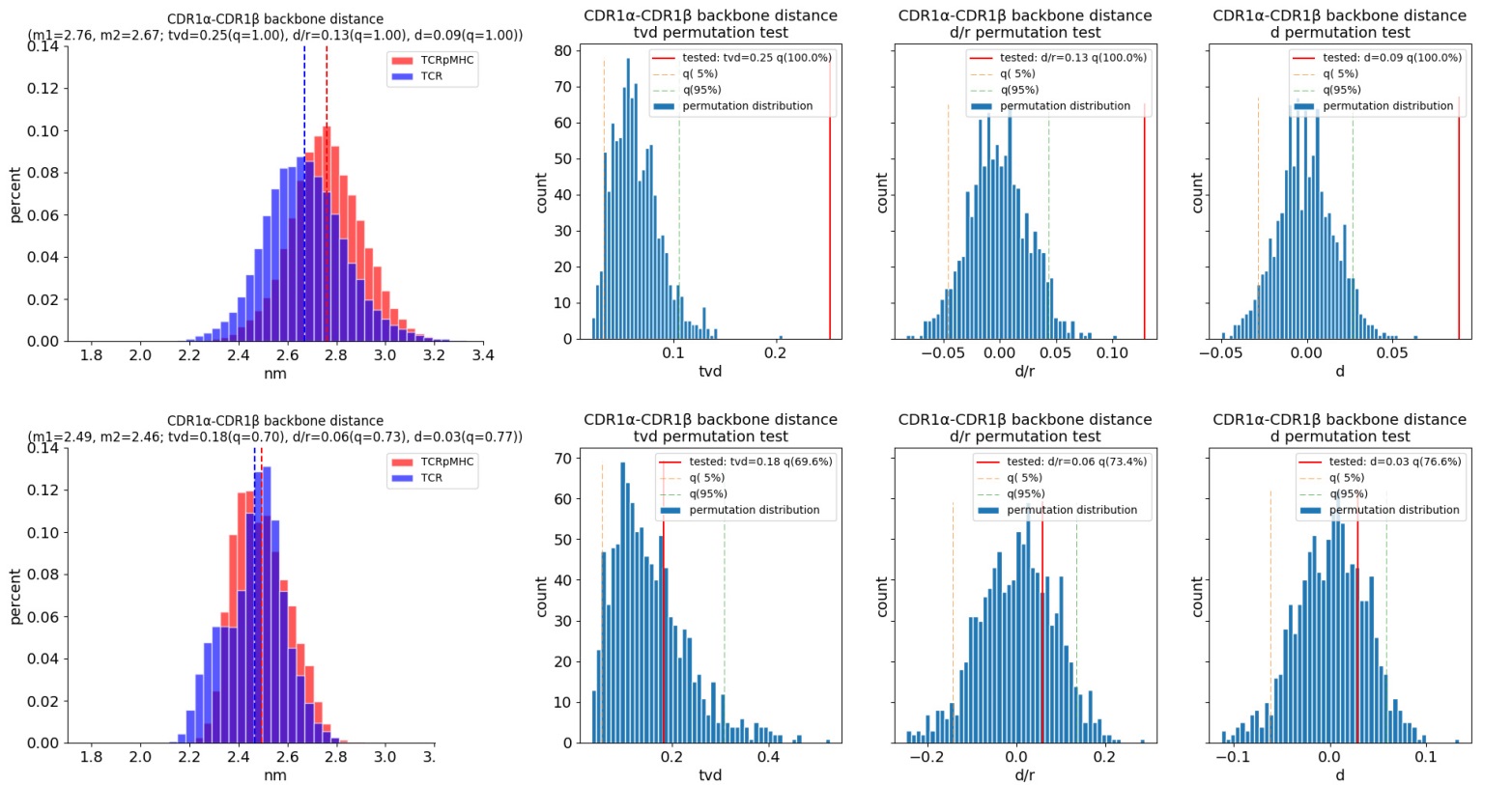


Figure S 2: Permutation test explanation for a significant difference in CDR1 loop distance (LC13 TCR; top) and non-significant difference (JM22 TCR; bottom). Left: distribution of values. Right: distribution of the permutation tests.
