# Supplementary material for: MHC binding affects the dynamics of different T-cell receptors in different ways
Source: PLoS Comput Biol. 2019 Sep 9;15(9):e1007338. doi: 10.1371/journal.pcbi.1007338 (PMC6752857; doi:10.1371/journal.pcbi.1007338)
Supplement: S3 Fig — RMSF and B-factors where normalised by subtracting the mean and dividing by the standard deviation in order to be on the same scale for plotting. This does not change the value of the correlation coefficient given in the title of the plots. (A) LC13 TCR, (B) JM22 TCR, (C) A6 TCR, (D) 1G4 TCR. (DOCX) [file pcbi.1007338.s003.docx]

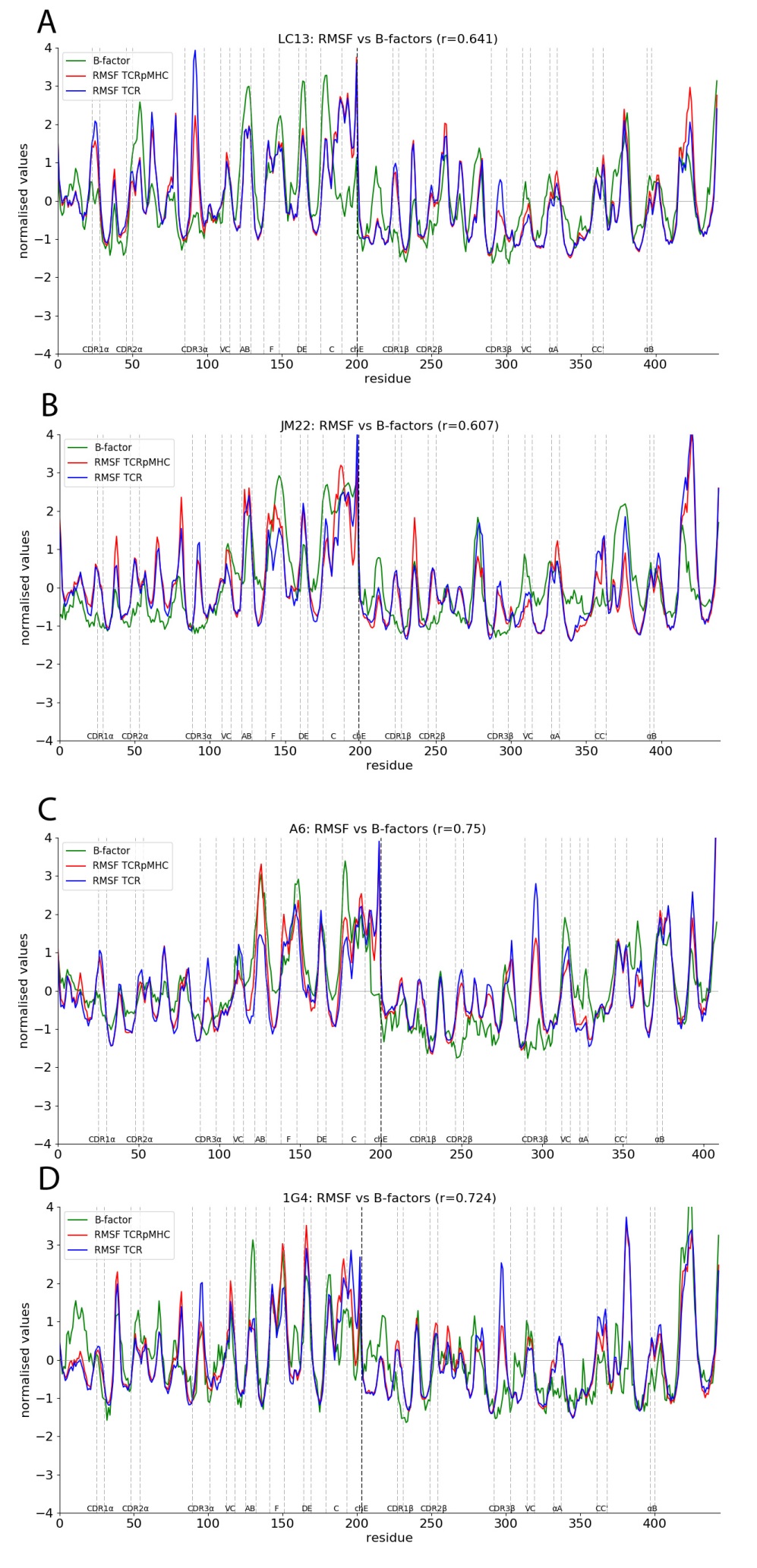


**Figure S 3: Comparison between RMSF values of the simulations and experimental B-factors. RMSF and B-factors where normalised by subtracting the mean and dividing by the standard deviation in order to be on the same scale for plotting. This does not change the value of the correlation coefficient given in the title of the plots.**
