# Supplementary material for: MHC binding affects the dynamics of different T-cell receptors in different ways
Source: PLoS Comput Biol. 2019 Sep 9;15(9):e1007338. doi: 10.1371/journal.pcbi.1007338 (PMC6752857; doi:10.1371/journal.pcbi.1007338)
Supplement: S1 Table — (DOCX) [file pcbi.1007338.s004.docx]

| **AB loop (Beddoe, 2009)** | **chain** | **start** | **end** | **length** | **comment** |
| --- | --- | --- | --- | --- | --- |
| LC13 | α | 129 | 136 | 8 |  |
| 1G4 | α | 127 | 134 | 8 |  |
| A6 | α | 123 | 130 | 8 |  |
| JM22 | α | 124 | 131 | 8 |  |
|  |  |  |  |  |  |
| **αV-C linker** |  |  |  |  |  |
| LC13 | α | 116 | 122 | 7 |  |
| 1G4 | α | 114 | 120 | 7 |  |
| A6 | α | 110 | 116 | 7 |  |
| JM22 | α | 111 | 117 | 7 |  |
|  |  |  |  |  |  |
| **βV-C linker (Brazin, 2015)** |  |  |  |  |  |
| LC13 | β | 118 | 123 | 6 |  |
| 1G4 | β | 113 | 118 | 6 |  |
| A6 | β | 117 | 122 | 6 |  |
| JM22 | β | 115 | 120 | 6 |  |
|  |  |  |  |  |  |
| **αA (He, 2015)** |  |  |  |  |  |
| LC13 | β | 136 | 141 | 6 |  |
| 1G4 | β | 131 | 136 | 6 |  |
| A6 | β | 128 | 147 | 6 | 14 AA deletion |
| JM22 | β | 133 | 138 | 6 |  |
|  |  |  |  |  |  |
| **αΒ (He, 2015)** |  |  |  |  |  |
| LC13 | β | 201 | 204 | 4 |  |
| 1G4 | β | 196 | 199 | 4 |  |
| A6 | β | 200 | 203 | 4 |  |
| JM22 | β | 198 | 201 | 4 |  |
|  |  |  |  |  |  |
| **F-strand Cα (Natarajan, 2016)** |  |  |  |  |  |
| LC13 | α | 183 | 197 | 15 |  |
| 1G4 | α | 181 | 195 | 15 |  |
| A6 | α | 177 | 191 | 15 |  |
| JM22 | α | 178 | 192 | 15 |  |
|  |  |  |  |  |  |
| **C-strand Cα (Natarajan, 2016)** |  |  |  |  |  |
| LC13 | α | 145 | 155 | 11 |  |
| 1G4 | α | 143 | 153 | 11 |  |
| A6 | α | 139 | 149 | 11 |  |
| JM22 | α | 140 | 150 | 11 |  |
|  |  |  |  |  |  |
| **DE-strand Cα (Kuhns, 2007)** |  |  |  |  |  |
| LC13 | α | 168 | 173 | 6 |  |
| 1G4 | α | 166 | 171 | 6 |  |
| A6 | α | 162 | 167 | 6 |  |
| JM22 | α | 163 | 168 | 6 |  |
|  |  |  |  |  |  |
| **CC’-strand Cβ (Kuhns, 2007)** |  |  |  |  |  |
| LC13 | β | 165 | 172 | 8 |  |
| 1G4 | β | 160 | 167 | 8 |  |
| A6 | β | 164 | 171 | 8 |  |
| JM22 | β | 162 | 169 | 8 |  |

Table S 1: Regions of interest of the four TCRs based on superimposition.
