# Supplementary material for: MHC binding affects the dynamics of different T-cell receptors in different ways
Source: PLoS Comput Biol. 2019 Sep 9;15(9):e1007338. doi: 10.1371/journal.pcbi.1007338 (PMC6752857; doi:10.1371/journal.pcbi.1007338)
Supplement: S2 Table — (DOCX) [file pcbi.1007338.s005.docx]

|  | LC13 | JM22 | A6 | 1G4 |  | LC13 | JM22 | A6 | 1G4 |  | LC13 | JM22 | A6 | 1G4 |
| --- | --- | --- | --- | --- | --- | --- | --- | --- | --- | --- | --- | --- | --- | --- |
|  | dr | dr | dr | dr |  | d | d | d | d |  | tvd | tvd | tvd | tvd |
| DIST CDR1 | 0,13 | 0,06 | 0,01 | 0,07 |  | 0,09 | 0,03 | 0,01 | 0,04 |  | 0,25 | 0,18 | 0,31 | 0,14 |
| DIST CDR2 | 0,06 | -0,08 | 0,19 | 0,17 |  | 0,04 | -0,04 | 0,14 | 0,12 |  | 0,11 | 0,12 | 0,37 | 0,31 |
| DIST CDR3 | 0,16 | 0,15 | -0,08 | -0,04 |  | 0,07 | 0,04 | -0,03 | -0,02 |  | 0,38 | 0,31 | 0,38 | 0,28 |
|  |  |  |  |  |  |  |  |  |  |  |  |  |  |  |
| RG CDR1a | 0,03 | -0,01 | -0,01 | 0,06 |  | 0,01 | 0,00 | 0,00 | 0,00 |  | 0,13 | 0,03 | 0,15 | 0,19 |
| RG CDR1b | 0,01 | -0,10 | 0,06 | 0,09 |  | 0,00 | -0,01 | 0,01 | 0,01 |  | 0,05 | 0,17 | 0,14 | 0,14 |
| RG CDR2a | -0,06 | 0,03 | 0,12 | 0,02 |  | 0,00 | 0,00 | 0,01 | 0,00 |  | 0,14 | 0,07 | 0,23 | 0,07 |
| RG CDR2b | -0,13 | 0,14 | 0,08 | -0,04 |  | -0,01 | 0,01 | 0,01 | 0,00 |  | 0,25 | 0,24 | 0,18 | 0,15 |
| RG CDR3a | -0,23 | -0,18 | 0,26 | 0,33 |  | -0,03 | -0,02 | 0,03 | 0,03 |  | 0,42 | 0,35 | 0,55 | 0,58 |
| RG CDR3b | -0,20 | -0,15 | -0,05 | 0,13 |  | -0,01 | -0,01 | 0,00 | 0,01 |  | 0,41 | 0,29 | 0,08 | 0,28 |
|  |  |  |  |  |  |  |  |  |  |  |  |  |  |  |
| RG ABloop | -0,01 | 0,10 | 0,05 | 0,04 |  | 0,00 | 0,01 | 0,01 | 0,01 |  | 0,02 | 0,16 | 0,13 | 0,11 |
| RG VCAlinker | -0,03 | -0,04 | 0,05 | -0,02 |  | 0,00 | 0,00 | 0,01 | 0,00 |  | 0,04 | 0,07 | 0,32 | 0,07 |
| RG VCBlinker | 0,04 | -0,21 | 0,03 | -0,11 |  | 0,00 | -0,03 | 0,00 | -0,02 |  | 0,08 | 0,38 | 0,08 | 0,21 |
| RG AlphaA | 0,03 | 0,00 | 0,08 | -0,04 |  | 0,00 | 0,00 | 0,00 | 0,00 |  | 0,04 | 0,08 | 0,14 | 0,09 |
| RG AlphaB | 0,01 | 0,00 | -0,06 | 0,03 |  | 0,00 | 0,00 | -0,01 | 0,00 |  | 0,02 | 0,04 | 0,17 | 0,05 |
| RG C-strand | 0,03 | -0,02 | -0,03 | 0,06 |  | 0,00 | 0,00 | -0,01 | 0,01 |  | 0,06 | 0,09 | 0,12 | 0,14 |
| RG F-strand | 0,04 | -0,03 | -0,09 | 0,07 |  | 0,01 | -0,01 | -0,02 | 0,02 |  | 0,08 | 0,20 | 0,16 | 0,16 |
| RG DE-strand | 0,01 | 0,00 | -0,12 | -0,11 |  | 0,00 | 0,00 | -0,02 | -0,02 |  | 0,04 | 0,10 | 0,34 | 0,21 |
| RG CC-strand | 0,01 | -0,01 | 0,01 | -0,02 |  | 0,00 | 0,00 | 0,00 | 0,00 |  | 0,01 | 0,04 | 0,08 | 0,04 |
|  |  |  |  |  |  |  |  |  |  |  |  |  |  |  |
| SASA CDR1a noMHC | 0,02 | 0,11 | 0,22 | 0,07 |  | 0,05 | 0,15 | 0,57 | 0,11 |  | 0,05 | 0,20 | 0,41 | 0,22 |
| SASA CDR1b noMHC | 0,01 | 0,01 | -0,10 | -0,15 |  | 0,02 | 0,02 | -0,20 | -0,32 |  | 0,02 | 0,04 | 0,19 | 0,26 |
| SASA CDR2a noMHC | 0,00 | 0,03 | 0,11 | 0,00 |  | 0,00 | 0,05 | 0,21 | -0,01 |  | 0,04 | 0,08 | 0,19 | 0,03 |
| SASA CDR2b noMHC | 0,01 | 0,03 | -0,04 | 0,01 |  | 0,02 | 0,07 | -0,06 | 0,01 |  | 0,11 | 0,06 | 0,07 | 0,10 |
| SASA CDR3a noMHC | -0,04 | -0,09 | 0,20 | 0,35 |  | -0,13 | -0,18 | 0,57 | 1,37 |  | 0,10 | 0,15 | 0,34 | 0,58 |
| SASA CDR3b noMHC | 0,01 | 0,03 | 0,07 | 0,12 |  | 0,02 | 0,09 | 0,22 | 0,29 |  | 0,03 | 0,07 | 0,13 | 0,23 |
| SASA CDR1a | -0,44 | -0,31 | -0,46 | -0,34 |  | -2,10 | -0,72 | -1,88 | -0,68 |  | 0,86 | 0,61 | 0,84 | 0,70 |
| SASA CDR1b | -0,27 | -0,21 | -0,11 | -0,48 |  | -0,54 | -0,64 | -0,25 | -1,63 |  | 0,59 | 0,43 | 0,22 | 0,90 |
| SASA CDR2a | -0,43 | -0,24 | -0,48 | -0,28 |  | -1,00 | -0,51 | -1,61 | -0,74 |  | 0,85 | 0,52 | 0,89 | 0,54 |
| SASA CDR2b | -0,36 | -0,49 | -0,13 | -0,43 |  | -1,00 | -1,84 | -0,27 | -1,45 |  | 0,72 | 0,93 | 0,28 | 0,79 |
| SASA CDR3a | -0,45 | -0,52 | -0,42 | -0,39 |  | -2,45 | -1,92 | -1,66 | -1,67 |  | 0,88 | 0,92 | 0,85 | 0,77 |
| SASA CDR3b | -0,53 | -0,45 | -0,50 | -0,48 |  | -2,62 | -2,25 | -2,76 | -2,18 |  | 0,97 | 0,89 | 0,96 | 0,95 |
|  |  |  |  |  |  |  |  |  |  |  |  |  |  |  |
| SASA ABloop | 0,00 | 0,06 | 0,15 | 0,02 |  | 0,00 | 0,18 | 0,54 | 0,06 |  | 0,02 | 0,13 | 0,26 | 0,05 |
| SASA VCAlinker | -0,01 | 0,02 | -0,15 | 0,01 |  | -0,02 | 0,04 | -0,43 | 0,02 |  | 0,03 | 0,05 | 0,29 | 0,04 |
| SASA VCBlinker | 0,03 | -0,07 | -0,04 | -0,06 |  | 0,07 | -0,18 | -0,10 | -0,16 |  | 0,06 | 0,21 | 0,10 | 0,10 |
| SASA AlphaA | -0,02 | -0,01 | 0,09 | -0,03 |  | -0,05 | -0,02 | 0,19 | -0,06 |  | 0,04 | 0,11 | 0,16 | 0,07 |
| SASA AlphaB | 0,00 | -0,05 | 0,06 | -0,05 |  | 0,00 | -0,06 | 0,19 | -0,05 |  | 0,02 | 0,09 | 0,10 | 0,09 |
| SASA C-strand | -0,02 | -0,03 | -0,08 | -0,03 |  | -0,09 | -0,12 | -0,34 | -0,13 |  | 0,04 | 0,20 | 0,14 | 0,08 |
| SASA F-strand | 0,03 | 0,09 | 0,04 | 0,09 |  | 0,09 | 0,34 | 0,17 | 0,38 |  | 0,05 | 0,20 | 0,11 | 0,17 |
| SASA DE-strand | 0,00 | -0,07 | -0,08 | -0,02 |  | 0,01 | -0,22 | -0,38 | -0,08 |  | 0,02 | 0,14 | 0,17 | 0,08 |
| SASA CC-strand | 0,01 | 0,01 | -0,09 | 0,00 |  | 0,01 | 0,03 | -0,28 | -0,01 |  | 0,02 | 0,09 | 0,23 | 0,05 |
|  |  |  |  |  |  |  |  |  |  |  |  |  |  |  |
| RMSF CDR1a | -0,13 | -0,12 | 0,00 | -0,16 |  | -0,03 | -0,02 | 0,00 | -0,02 |  | 0,27 | 0,24 | 0,28 | 0,38 |
| RMSF CDR2a | -0,10 | -0,14 | -0,03 | -0,08 |  | -0,02 | -0,02 | -0,01 | -0,01 |  | 0,18 | 0,30 | 0,25 | 0,29 |
| RMSF CDR3a | -0,12 | -0,13 | -0,06 | -0,15 |  | -0,05 | -0,03 | -0,01 | -0,04 |  | 0,21 | 0,31 | 0,27 | 0,22 |
| RMSF CDR1b | -0,12 | -0,19 | 0,00 | -0,05 |  | -0,02 | -0,03 | 0,00 | -0,01 |  | 0,23 | 0,30 | 0,34 | 0,22 |
| RMSF CDR2b | -0,07 | -0,14 | -0,05 | -0,04 |  | -0,01 | -0,03 | -0,01 | -0,01 |  | 0,16 | 0,35 | 0,27 | 0,23 |
| RMSF CDR3b | -0,11 | -0,18 | -0,07 | -0,11 |  | -0,02 | -0,03 | -0,03 | -0,04 |  | 0,21 | 0,34 | 0,24 | 0,26 |
|  |  |  |  |  |  |  |  |  |  |  |  |  |  |  |
| RMSF ABloop | -0,02 | -0,07 | 0,20 | -0,10 |  | -0,01 | -0,02 | 0,10 | -0,02 |  | 0,11 | 0,39 | 0,40 | 0,24 |
| RMSF VC_Alinker | 0,00 | -0,03 | -0,06 | -0,01 |  | 0,00 | -0,01 | -0,01 | 0,00 |  | 0,08 | 0,39 | 0,34 | 0,33 |
| RMSF VC_Blinker | 0,08 | -0,26 | -0,02 | -0,03 |  | 0,01 | -0,03 | -0,01 | 0,00 |  | 0,15 | 0,47 | 0,33 | 0,22 |
| RMSF AlphaA | 0,06 | -0,01 | 0,13 | -0,07 |  | 0,01 | 0,00 | 0,02 | -0,01 |  | 0,15 | 0,37 | 0,42 | 0,37 |
| RMSF AlphaB | 0,06 | -0,17 | 0,05 | -0,14 |  | 0,01 | -0,03 | 0,02 | -0,02 |  | 0,09 | 0,30 | 0,48 | 0,33 |
| RMSF C-strand | -0,01 | -0,04 | 0,12 | -0,02 |  | 0,00 | -0,01 | 0,04 | -0,01 |  | 0,07 | 0,24 | 0,35 | 0,17 |
| RMSF F-strand | -0,03 | 0,04 | 0,05 | 0,00 |  | -0,01 | 0,01 | 0,02 | 0,00 |  | 0,12 | 0,29 | 0,45 | 0,27 |
| RMSF DE-strand | 0,01 | -0,08 | 0,01 | -0,01 |  | 0,00 | -0,03 | 0,00 | 0,00 |  | 0,10 | 0,28 | 0,45 | 0,25 |
| RMSF CC-strand | 0,00 | -0,19 | 0,03 | -0,18 |  | 0,00 | -0,05 | 0,01 | -0,04 |  | 0,08 | 0,49 | 0,50 | 0,53 |
|  |  |  |  |  |  |  |  |  |  |  |  |  |  |  |
| n of H-bonds | -0,04 | -0,09 | 0,04 | -0,03 |  | -0,51 | -1,37 | 0,61 | -0,41 |  | 0,06 | 0,16 | 0,06 | 0,07 |

Table S 2: Same as Table 2 but showing and colouring also non significant values.
